# Exploring galectin-3’s role in predicting mild cognitive impairment in type 2 diabetes and its regulation by miRNAs

**DOI:** 10.3389/fmed.2024.1443133

**Published:** 2024-07-31

**Authors:** Xueling Zhou, Ning Dai, Dandan Yu, Tong Niu, Shaohua Wang

**Affiliations:** ^1^School of Medicine, Southeast University, Nanjing, China; ^2^Department of Endocrinology, Affiliated Zhongda Hospital of Southeast University, Nanjing, China; ^3^Department of ENT, Maanshan People’s Hospital, Maanshan, China

**Keywords:** mild cognitive impairment, type 2 diabetes mellitus, galectin-3, nomogram, miRNA128

## Abstract

**Objective:**

This study aimed to investigate the role of galectin-3 (Gal-3; coded by LGALS3 gene), as a biomarker for MCI in T2DM patients and to develop and validate a predictive nomogram integrating galectin-3 with clinical risk factors for MCI prediction. Additionally, microRNA regulation of LGALS3 was explored.

**Methods:**

The study employed a cross-sectional design. A total of 329 hospitalized T2DM patients were recruited and randomly allocated into a training cohort (*n* = 231) and a validation cohort (*n* = 98) using 7:3 ratio. Demographic data and neuropsychological assessments were recorded for all participants. Plasma levels of galectin-3 were measured using ELISA assay. We employed Spearman’s correlation and multivariable linear regression to analyze the relationship between galectin-3 levels and cognitive performance. Furthermore, univariate and multivariate logistic regression analyses were conducted to identify independent risk factors for MCI in T2DM patients. Based on these analyses, a predictive nomogram incorporating galectin-3 and clinical predictors was developed. The model’s performance was evaluated in terms of discrimination, calibration, and clinical utility. Regulatory miRNAs were identified using bioinformatics and their interactions with LGALS3 were confirmed through qRT-PCR and luciferase reporter assays.

**Results:**

Galectin-3 was identified as an independent risk factor for MCI, with significant correlations to cognitive decline in T2DM patients. The developed nomogram, incorporating Gal-3, age, and education levels, demonstrated excellent predictive performance with an AUC of 0.813 in the training cohort and 0.775 in the validation cohort. The model outperformed the baseline galectin-3 model and showed a higher net benefit in clinical decision-making. Hsa-miR-128-3p was significantly downregulated in MCI patients, correlating with increased Gal-3 levels, while Luciferase assays confirmed miR-128-3p’s specific binding and influence on LGALS3.

**Conclusion:**

Our findings emphasize the utility of Gal-3 as a viable biomarker for early detection of MCI in T2DM patients. The validated nomogram offers a practical tool for clinical decision-making, facilitating early interventions to potentially delay the progression of cognitive impairment. Additionally, further research on miRNA128’s regulation of Gal-3 levels is essential to substantiate our results.

## 1 Introduction

Type 2 diabetes mellitus (T2DM) is the predominant form of diabetes, representing over 90% of all diabetes cases (1). T2DM is intricately linked to cognitive impairment, manifesting as declines in comprehension, language skills, judgment, orientation, logical reasoning, learning capacity, and memory (2). In the early stages, this cognitive decline is classified as mild cognitive impairment (MCI), which could progress to dementia and severely impact the quality of life of patients (3, 4). Research indicates that diabetes could hasten this progression (5–7), emphasizing the necessity for early prevention and treatment of cognitive impairment in T2DM patients. Although MCI represents the optimal period for early detection and intervention, the lack of specific clinical symptoms and effective early screening methods poses significant challenges. Consequently, identifying sensitive detection methods for MCI in T2DM patients is crucial for providing a robust foundation for early diagnosis and intervention.

Galectin-3 (Gal-3), encoded by LGALS3 gene, is a multifaceted lectin with an N-terminal domain that modulates its cellular functions, an α-collagen-like sequence, and a C-terminal carbohydrate recognition domain (CRD) that binds to β-galactoside (8, 9). As a member of the 15-member lectin family, Galectin-3 is distinguished by its galactose-binding domains and is extensively expressed in various cell types, including macrophages, natural killer cells, T and B lymphocytes, neutrophils, and eosinophils, playing a crucial role in the immune response (10, 11). Notably, Galectin-3 is highly expressed in macrophages and microglia, the resident macrophages of the central nervous system, with its expression significantly upregulated by inflammatory stimuli (12). Galectin-3 contributes to diabetic complications by serving as a receptor for AGEs and ALEs, promoting inflammation which leads to insulin resistance. Additionally, it disrupts insulin receptor signaling, exacerbating insulin resistance and glucose intolerance (13–15). Insulin resistance and neuroinflammation are proposed as the primary mechanisms of cognitive impairment associated with diabetes (16, 17). MicroRNAs (miRNAs) are small RNA fragments, approximately 21–23 nucleotides in length, which regulate gene expression by binding to the 3′ untranslated region (3’-UTR) or coding domain sequences (CDSs) of target messenger RNAs (mRNAs) (18, 19). miRNAs have widely existed *in vivo*, and were implicated in the post-transcription regulation of gene expression by repressing mRNA translation or inhibiting mRNA and protein degradation (20, 21). Additionally, peripheral miRNAs are stable and easily accessible, reflecting the dynamic changes observed in central nervous system miRNAs. For example, in Alzheimer’s disease (AD) patients, miRNAs exhibit consistent changes in both peripheral body fluids and postmortem brain tissues (22). Given the limited studies on the miRNA regulation of LGALS3, analyzing miRNAs that regulate LGALS3 through bioinformatics methods and validating their targeted regulatory effects could provide a more comprehensive understanding of Galectin-3 expression trends in both peripheral and central systems. Therefore, designing a method that is not only feasible and simple but also accurately distinguishes MCI in T2DM with high calibration is crucial.

Nomograms rely on user-friendly digital interfaces and intuitive visualizations, enabling a more comprehensive, multi-dimensional integration of risk factors. They also allow for the visualization and graphical representation of logistic regression analysis results. This greatly enhances predictive accuracy and facilitates user interpretation, thereby maximally aiding medical personnel in clinical decision-making (23). Currently, there are few studies applying nomogram models to predict MCI in patients with T2DM. Therefore, the aim of this study was to develop and validate a nomogram that incorporated both Galectin-3 and clinicopathologic risk factors for individual prediction of MCI in patients with T2DM.

## 2 Materials and methods

### 2.1 Study design and participants

**Figure 1:**
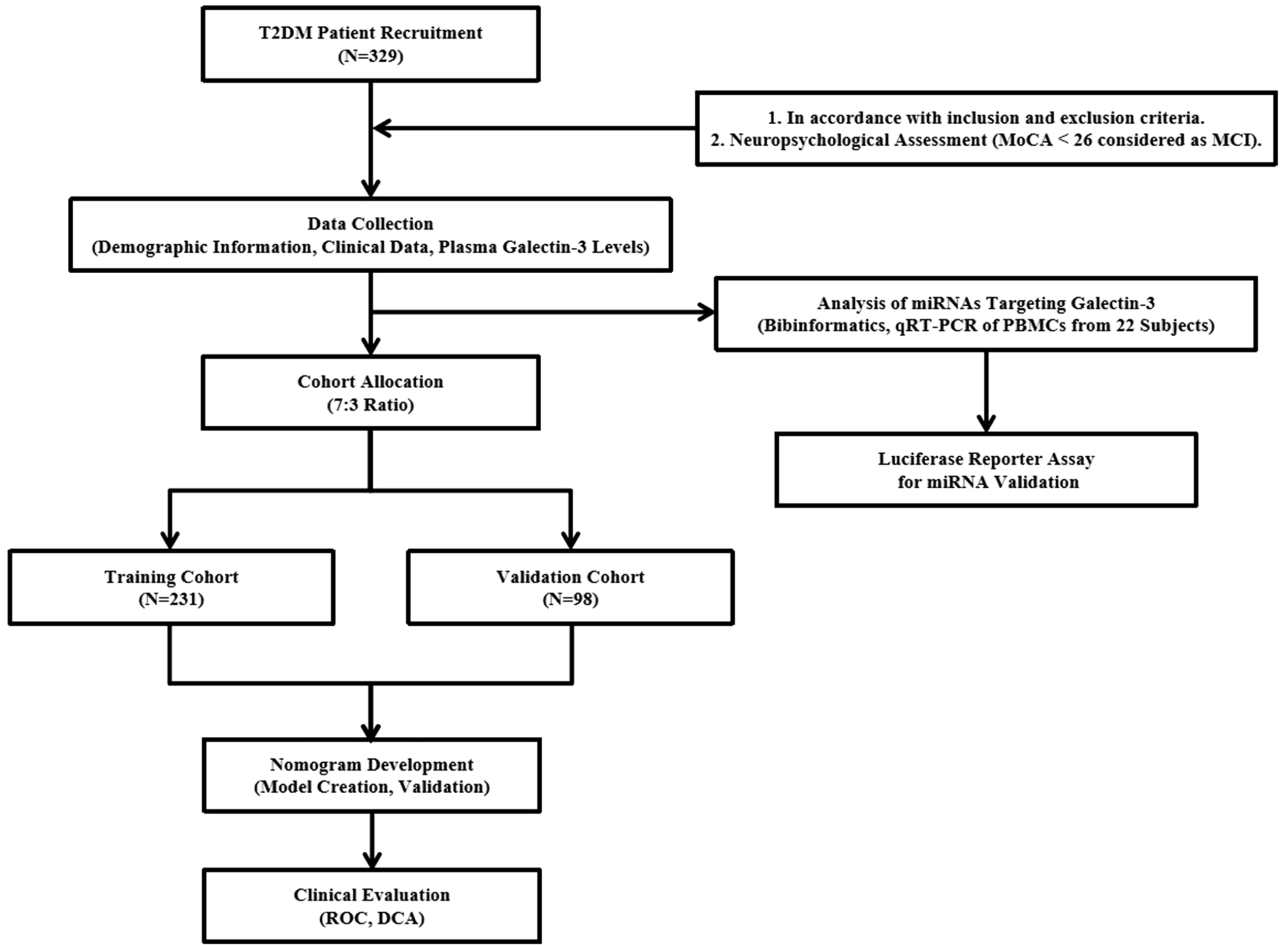
The whole analysis flow for this study.

The study workflow in Figure 1 illustrates the research process.

Between August 2022 and May 2023, a total of 329 patients aged 40 to 75 with Type 2 diabetes mellitus (T2DM) were enrolled from the Department of Endocrinology at the Affiliated Zhongda Hospital, Southeast University. The study used cross-sectional design. Eligibility criteria included a minimum of 3 years since T2DM diagnosis and at least 6 years of education. Patients were excluded if they had acute diabetic complications (e.g., ketoacidosis, severe hypoglycemia, cardiovascular crises), neurological disorders, substance misuse (including alcohol and drugs), other serious health conditions (such as thyroid dysfunction, severe infections, recent surgeries, or malignancies) that could affect cognitive function, or sensory impairments (deafness or visual issues) that could compromise neuropsychometric testing. Cognitive function was assessed using the Montreal Cognitive Assessment (MoCA), with an additional point added to scores under 30 for participants with less than 12 years of education. Patients were classified into either the mild cognitive impairment (MCI) group (MoCA < 26) or the normal control group (NC) group (MoCA ≥ 26), with 185 individuals in NC group and 144 individuals in MCI group. This study received approval from the institutional Research Ethics Committee (approval no. 2023ZDSYLL435-P01).

### 2.2 Clinical data collection

Participant demographic and clinical data including age, gender, educational background, and diabetes status were collected. Anthropometric measurements to determine body mass index (BMI) were based on recorded weights and heights. Fasting overnight, blood samples were drawn by trained nurses, with plasma separated and preserved at −80°C. The Center Laboratory conducted analyses of fasting plasma glucose (FPG), glycosylated hemoglobin (HbA1c), triglycerides (TG), total cholesterol (TC), blood urea nitrogen (BUN), creatinine (Cr), and both high-density and low-density lipoprotein cholesterol (HDL-C and LDL-C). Data extraction was performed from medical records. Plasma galectin-3 levels were quantified using enzyme-linked immunosorbent assay (ELISA) kits (BYabscience, China, Catalog No.:BY-EH180582), according to the manufacturer’s instructions. Following the manufacturer’s guidelines.

### 2.3 Human neuropsychological tests

All participants underwent a series of neuropsychological tests to evaluate various cognitive functions. The Montreal Cognitive Assessment (MoCA) (24, 25) was used to assess global cognitive abilities and to detect cognitive impairments in the participants, while the Mini-Mental State Examination (MMSE) (25, 26) was employed to further evaluate cognitive functions.

Information processing speed was measured via the Trail Making Test-A (TMTA) (27), assessing attention and executive function with a maximum non-completion score of 150 s. Executive functions were further evaluated using the Digit Span Test (DST) (28), which focuses on immediate memory and attention; the Verbal Fluency Test (VFT) (29), assessing language abilities and executive functions; and the Trail Making Test-B (TMTB) (27), measuring executive function with a maximum non-completion score of 300 s. Memory functions were evaluated through the Auditory Verbal Learning Test-Immediate Recall (AVLT-IR) and Delayed Recall (AVLT-DR) (30), assessing immediate and delayed memory functions, and the Logical Memory Test (LMT) (31) was used to gauge scene memory function by testing recall of logical sequences and scene memory. Additionally, the Clock Drawing Test (CDT) was used to measure abstract thinking, spatial–temporal orientation, and executive function (32), scored out of 4 based on the outline, number placement, and the lengths and positions of the hands drawn. These domain-specific tests involving memory, executive function, visuospatial abilities, language, and processing speed are also important adjuncts in the screening for MCI.

### 2.4 Quantitative real-time polymerase chain reaction

Among the participants, partial whole-blood samples from 22 subjects, including 11 from the control group and 11 from the MCI group, were utilized for isolating human peripheral blood mononuclear cells (PBMCs) through Ficoll-Paque density gradient separation (density 1.077 g/dL, TBD Science, China, LTS1077).

Total RNA was extracted from the isolated PBMC samples using TRIzol reagent (Invitrogen, Carlsbad, CA, United States) following the manufacturer’s instructions, and the quantity of the extracted RNA was determined using a NanoDrop spectrophotometer (Thermo Scientific Nanodrop 2000).

For miRNA RT-qPCR, cDNA stem-loop RT primers were used to produce cDNA for specific miRNAs (33, 34). Extracted RNA was converted to cDNA by PrimeScriptTM RT Master Mix (RR036A, Takara, Japan). And Real-time qPCR analysis was performed with TB GreenTM Premix Ex Taq IITM (RR820A, TaKaRa) on a Step ONE Plus Real time PCR system (Applied Biosystems). U6 served as the internal reference gene for miRNA, and the relative levels were calculated using the relative quantification (2^−ΔΔCt^) method (35). Sequences of the primers used for the analyses were shown in Table 1.

**Table 1:** Sequences of the primer pairs used to analyze the genes.

Gene name	Primer sequence (5′ → 3′)
hsa-miR-424-3p	RT: GTCGTATCCAGTGCAGGGTCCGAGGTATTCGCACTGGATACGACATAGCA
	F: CAAAACGTGAGGCGCTGCTAT
	R:ATCCAGTGCAGGGTCCGAGG
hsa-miR-744-5p	RT: GTCGTATCCAGTGCAGGGTCCGAGGTATTCGCACTGGATACGACTGCTGT
	F: CGTGCGGGGCTAGGGCTA
	R:ATCCAGTGCAGGGTCCGAGG
hsa-miR-128-3p	RT: GTCGTATCCAGTGCAGGGTCCGAGGTATTCGCACTGGATACGACAAAGAG
	F:GCTCACAGTGAACCGGTCTCTTT
	R:ATCCAGTGCAGGGTCCGAGG
U6	F:CTCGCTTCGGCAGCACA
	R:AACGCTTCACGAATTTGCGT

All miRNA sequences used in this study are listed in Table 1.

### 2.5 Dual-luciferase reporter assay

Dual-luciferase assays were performed in human embryonic kidney (HEK) 293 T cells.

The 3′untranslated region (UTR) sequence containing the *hsa-miRNA–*binding site or a mutated binding site were cloned into the pmirGLO dual-luciferase vector (pmirGLO vector) (19, 36), which contained both the firefly luciferase gene and the renilla luciferase gene (Supplementary Figure 1). HEK 293 T cells were cotransfected with pmirGLO vector containing a wild-type (WT) or mutated (MUT) sequence in multiple cloning site (MCS), and miRNA mimics or inhibitors (General Biosystems, China) by Lipofectamine 3,000 (ThermoFisher, L3000015), according to the manufacturer’s instructions. Reporter constructs were confirmed by sequencing (Supplementary Figure 2). Luciferase activity was measured 48 h after transfection of HEK-293Tcells using the Dual-Glo Luciferase Assay (Promega, E2920) according to the manufacturer’s protocol. Coexpressed Renilla luciferase on the pmirGLO vector was used as an internal control to normalize the firefly luciferase activity.

### 2.6 Statistical analysis

Statistical analysis was conducted using GraphPad Prism 8.0, SPSS 24.0, and R software (version 4.2.1). Stratified sampling techniques were used to minimize potential bias in model training and validation. Results for molecular experiments and clinical data are presented as mean ± SD or median and interquartile range (IQR). Student’s t-test was used for normally distributed variables, while the Mann–Whitney U test was used for asymmetrically distributed variables. The Chi-squared test was employed for binary variables. Spearman and partial correlation analyses were performed with or without adjustment for confounding factors. Multiple linear regression was used to assess the impact of factors on global cognitive function, and binary logistic analysis was used to identify MCI risk factors.

To prove the value of Gal-3 in MCI assessment, a nomogram was established based on the multivariable analysis results, and to maximize the predictive power of the model, we constructed it in the training cohort and assessed its accuracy and generalizability in the validation cohort.

In order to validate the calibration of the model, 500 bootstrap resampling techniques were employed, with an expectation that the detection would align closely with a 45° diagonal line (37). The clinical utility of the nomogram was evaluated through decision curve analysis (DCA) (38). Predictive models were established and evaluated using R software, and ROC curve analysis was conducted for diagnostic accuracy. All tests were two-tailed, and *p*-values < 0.05 were considered statistically significant.

## 3 Results

### 3.1 Comparison of clinical characteristics in T2DM patients with and without MCI

A total of 329 eligible patients were randomly assigned to a training cohort (*n* = 231) and a validation cohort (*n* = 98) at a ratio of 7:3. The prevalence of Mild Cognitive Impairment (MCI) was similar between the cohorts, with 43.72% in the training cohort and 43.88% in the validation cohort, resulting in no statistically significant difference (*p* = 1.0). Stratified sampling techniques were used to ensure similar distributions of demographic and clinical characteristics, minimizing potential bias in model training and validation (Table 2).

**Table 2:** Characteristics of patients in the training and validation cohorts.

	Training cohort			Validation cohort		
Characteristics	NC group	MCI group	*P*-value	NC group	MCI group	*P*-value
*n*	130	101		55	43	
Age (year)	57.5 (51.25, 64)	64 (59, 69)	<0.001	57.309 ± 8.4959	60.721 ± 7.2942	0.039
Gender, *n* (%)			0.079			0.002
Male	88 (38.1%)	57 (24.7%)		47 (48%)	25 (25.5%)	
Female	42 (18.2%)	44 (19%)		8 (8.2%)	18 (18.4%)	
Education (year)	12 (11, 15)	12 (9, 12)	<0.001	12 (11, 15)	12 (9, 12)	0.013
DM duration (year)	10 (5, 15)	10 (7, 20)	0.036	9 (5, 15)	12 (7, 16.5)	0.123
HTN, *n* (%)			0.023			0.819
No	71 (30.7%)	40 (17.3%)		23 (23.5%)	17 (17.3%)	
Yes	59 (25.5%)	61 (26.4%)		32 (32.7%)	26 (26.5%)	
HTN duration (year)	0 (0, 10)	5 (0, 15)	0.019	6 (0, 10)	4 (0, 10)	0.789
Smoking history, *n* (%)			0.227			0.839
No	87 (37.7%)	75 (32.5%)		27 (27.6%)	22 (22.4%)	
Yes	43 (18.6%)	26 (11.3%)		28 (28.6%)	21 (21.4%)	
Alcohol use, *n* (%)			0.472			0.139
No	109 (47.2%)	81 (35.1%)		36 (36.7%)	34 (34.7%)	
Yes	21 (9.1%)	20 (8.7%)		19 (19.4%)	9 (9.2%)	
BMI (Kg/m^2^)	24.653 ± 3.202	24.938 ± 3.0452	0.494	25.9 (23.25, 26.8)	24.6 (22.307, 26.5)	0.296
HbA1c (%)	8.34 (7.16, 9.78)	8.365 (7.57, 9.6725)	0.478	8.2287 ± 1.9733	8.674 ± 1.6348	0.242
FBG (mmol/L)	7.475 (5.825, 8.98)	7.24 (6.07, 9.08)	0.755	7 (5.955, 8.51)	7.8 (6.16, 9.34)	0.225
TG (mmol/L)	1.46 (1.0175, 1.9725)	1.32 (0.96, 1.87)	0.235	1.63 (0.975, 2.25)	1.22 (1.02, 2.01)	0.678
TC (mmol/L)	4.3969 ± 1.0556	4.5452 ± 1.1346	0.309	4.3202 ± 0.84028	4.2854 ± 1.0672	0.863
HDL-C (mmol/L)	1.05 (0.91, 1.215)	1.03 (0.9, 1.24)	0.949	0.97 (0.85, 1.15)	1.02 (0.89, 1.18)	0.345
LDL-C (mmol/L)	2.4857 ± 0.77108	2.5815 ± 0.86383	0.38	2.4935 ± 0.67331	2.2924 ± 0.89367	0.231
Cr (μmol/L)	69 (57, 80)	68 (53, 80)	0.589	69 (57.5, 84)	64 (59, 83.5)	0.524
BUN (mmol/L)	6.25 (5.1, 7.225)	6.6 (5.8, 7.9)	0.02	6.4 (5.6, 7.6)	6.2 (5.35, 7.5)	0.39
ALT (U/L)	17 (13, 24)	16 (13, 23)	0.285	17 (13, 22)	17 (10.5, 22.5)	0.696
AST (U/L)	17 (14, 22)	16 (13, 21)	0.141	17 (14, 19)	16 (12.5, 20.5)	0.749
Galectin-3 (ng/m)	5.0438 (4.3678, 6.07)	6.5104 (5.4374, 7.6501)	<0.001	5.1195 (4.1894, 5.9812)	6.8401 (5.3052, 7.3674)	<0.001
MOCA	28 (27, 29)	24 (23, 25)	<0.001	29 (28, 29)	24 (24, 25)	<0.001
MMSE	29 (28, 30)	27 (26, 28)	<0.001	29 (28, 30)	27 (25.5, 28)	<0.001
DST	12 (11, 14)	11 (9, 13)	<0.001	13 (11, 14)	11 (9, 12.75)	0.003
VFT	18 (16, 21)	16 (13.75, 19)	<0.001	18 (15, 24)	16 (12.5, 18)	<0.001
CDT	4 (3, 4)	3 (2, 4)	<0.001	4 (3, 4)	3 (2, 4)	0.011
TMTA	60 (48, 76.25)	67 (56, 88)	0.001	59 (48, 82)	67.5 (55, 78)	0.137
TMTB	133 (110.5, 164.75)	169.5 (120, 211.25)	<0.001	144 (115, 168)	164 (132, 190)	0.011
AVLT-IR	17.325 ± 5.6933	14.485 ± 5.1399	<0.001	17.906 ± 5.9104	15.732 ± 5.2584	0.067
AVLT-DR	6 (4, 9)	4 (2.75, 6.25)	<0.001	6 (3.75, 9.25)	5 (3, 7)	0.248
LMT	6 (4, 9)	4 (3, 8)	0.008	5 (3, 9)	4 (2, 7)	0.148

In the training cohort, MCI patients were older (64 [59, 69] vs. 57.5 [51.25, 64] years, *p* < 0.001), had a longer duration of diabetes (10 [7, 20] vs. 10 [5, 15] years, *p* = 0.036), a higher prevalence of hypertension (HTN; 26.4% vs. 25.5%, *p* = 0.023), and lower education levels (12 [9, 12] vs. 12 [11, 15] years, *p* < 0.001) compared to NC patients. The percentage of females was higher in the MCI group, but this difference was not statistically significant (*p* = 0.079).

In the validation cohort, MCI patients were older (60.721 ± 7.2942 vs. 57.309 ± 8.4959 years, *p* = 0.039) and had a higher percentage of females (18.4% vs. 8.2%, *p* = 0.002) compared to NC patients. However, in both the training and validation cohorts, all other relevant factors, including BMI, HbA1c, FPG, TG, TC, HDL-C, LDL-C, Cr, BUN, ALT, and AST, were well-matched between the two groups (all *p* > 0.05). Other characteristics, such as smoking and alcohol use, also showed no significant differences between the groups. MCI patients in both cohorts had significantly lower scores on the MoCA, MMSE, DST, VFT, and CDT tests, and took longer to complete the TMTB test compared to NC patients (all *p* < 0.05), as shown in Table 2.

### 3.2 Association between Gal-3 levels and cognitive function in T2DM patients

**Figure 2:**
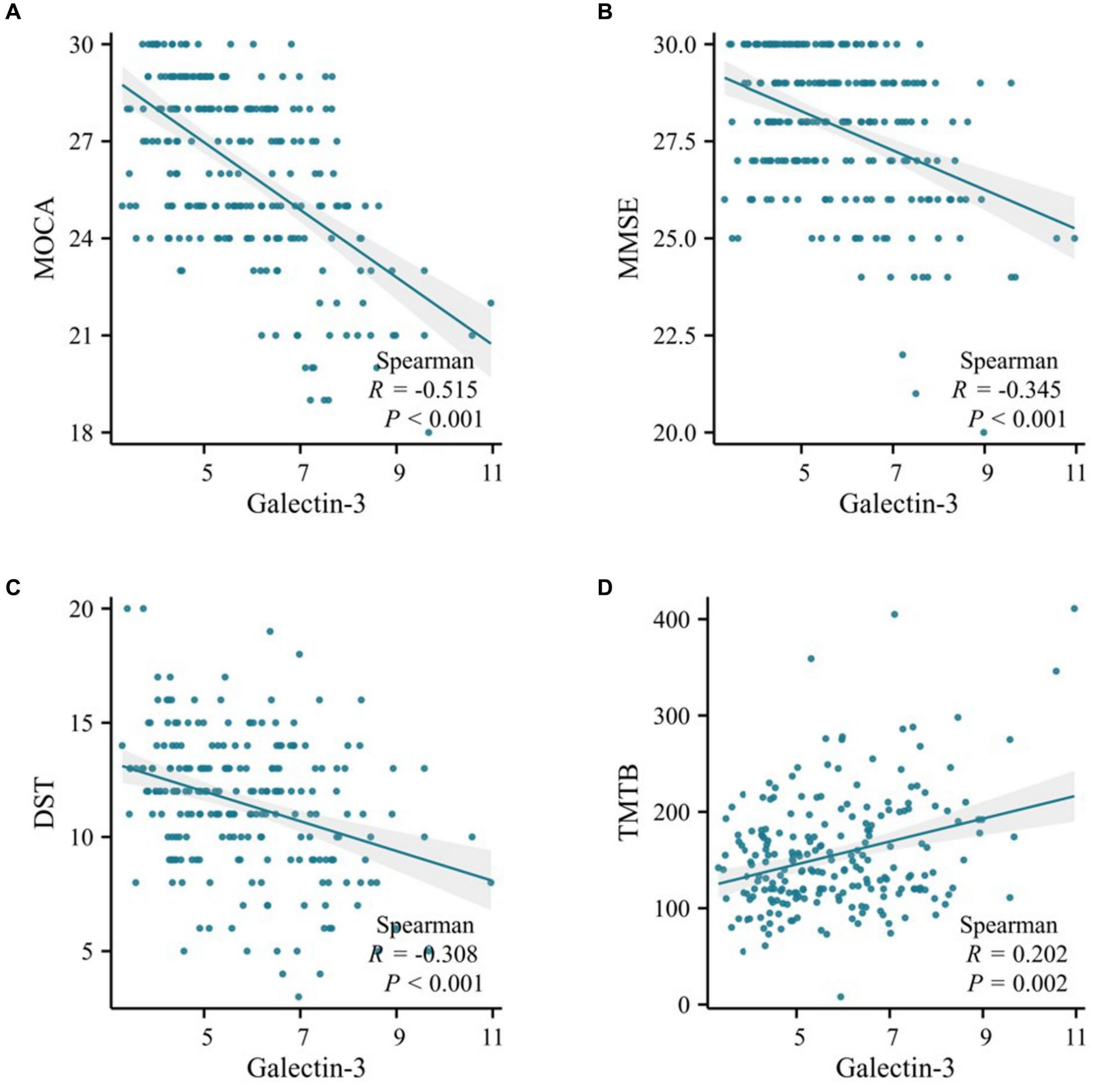
Scatter plot of correlation analysis between galectin-3 and cognitive function in the training cohort. **(A)** Spearman correlation analysis of Galectin-3 levels and MOCA scores. **(B)** Spearman correlation analysis of Galectin-3 levels and MMSE scores. **(C)** Spearman correlation analysis of Galectin-3 levels and DST scores. **(D)** Spearman correlation analysis of Galectin-3 levels and TMTB scores.

The study revealed that in both the training and validation cohorts, levels of Galectin-3 (Gal-3) were significantly higher in the MCI group of T2DM patients. This finding prompted an analysis of the relationship between Gal-3 levels and neuropsychometric test results. In the training cohort, Spearman correlation analysis identified a significant negative association between Gal-3 levels and both MOCA (R = -0.515, *p* < 0.001) and MMSE (R = -0.345, *p <* 0.001) scores (Figures 2A,B). Additionally, Gal-3 levels negatively correlated with DST (R = -0.308, *p* < 0.001) and positively with TMTB (R = 0.202, *p* = 0.002) (Figures 2C,D). Considering the variations in age, gender, education, hypertension prevalence, and diabetes duration between patients with and without MCI, partial association analyses were conducted. These analyses revealed that both the unadjusted and adjusted models consistently demonstrated a negative association of Gal-3 levels with MOCA, MMSE, DST, VFT, CDT, and AVLT-IR scores, and a positive association with TMTB scores (all *p* < 0.05; as detailed in Supplementary Table 1). The MMSE and MOCA tests were used to assess global cognitive function, DST evaluated memory and attention, VFT assessed language abilities and executive function, CDT measured praxis and planning abilities, and TMTB evaluated executive function. Additionally, stepwise multivariable linear regression was employed using MOCA, MMSE, DST, and TMTB scores as dependent variables to identify predictive factors for cognitive function. The model adjustments for potential confounders were stratified into three levels: Model 1 adjusted for age and gender, while Models 2 and 3 incrementally included adjustments for education, hypertension prevalence, and diabetes duration. Overall, these results underscored that Gal-3 independently contributed to the risk associated with cognitive function, as evaluated by MOCA, MMSE, DST, and TMTB scores (*p* < 0.001; Table 3).

**Table 3:** Multiple linear regression analysis of the association between galectin-3 and cognitive function in T2DM patients of the training cohort.

	Model 1		Model 2		Model 3	
Variable	Standardized β coefficient	*P*-value	Standardized β coefficient	*P*-value	Standardized β coefficient	*P*-value
MOCA	−0.538	<0.001	−0.513	< 0.001	−0.518	<0.001
MMSE	−0.373	<0.001	−0.348	< 0.001	−0.337	<0.001
DST	−0.322	<0.001	−0.315	< 0.001	−0.314	<0.001
TMTB	0.278	<0.001	0.239	< 0.001	0.244	<0.001

Model 1, Adjusted for Age and Gender; Model 2, Additionally adjusted for Age, Gender and Education; Model 3, Additionally adjusted for Age, Gender, Education, the prevalence of Hypertension and DM Duration.

### 3.3 Logistic regression analysis of risk factors for MCI in T2DM patients

**Figure 3:**
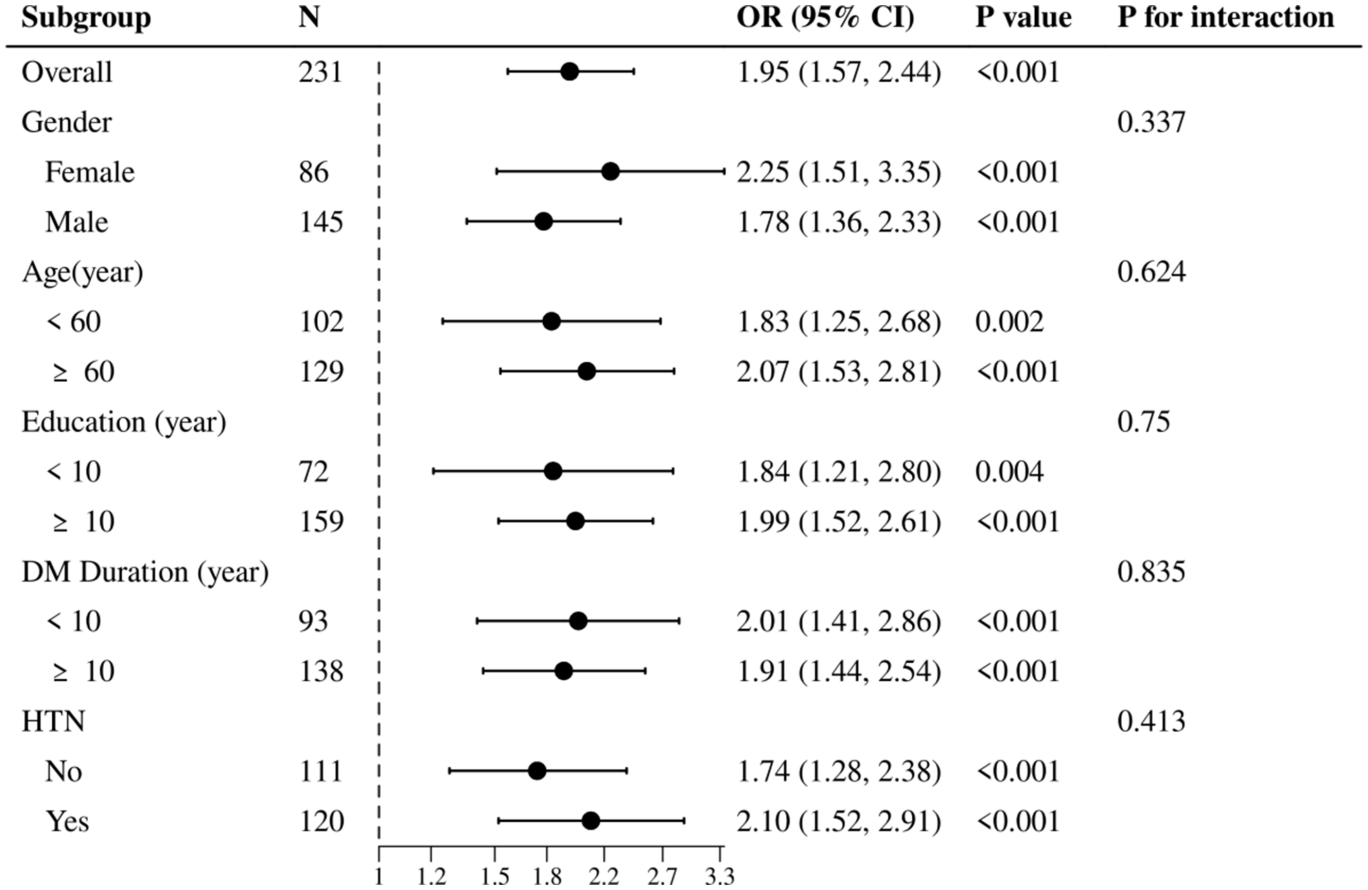
Subgroup analysis of galectin-3’s impact on MCI across key clinical factors.

To determine whether elevated levels of Galectin-3 serve as a risk factor for MCI in T2DM, logistic regression was employed to assess multiple variables jointly for independent association with binary outcomes. Both univariate and multivariate logistic regression analyses were conducted to explore the relationship between various factors and MCI in T2DM patients. As shown in Table 4, univariate logistic regression analysis identified that age, education, prevalence of hypertension, and DM duration were significantly associated with the prediction of MCI (*p* < 0.05). Multivariate logistic regression analysis further revealed that age, education, and Galectin-3 levels were independent predictors of MCI in T2DM patients (p < 0.05). The results indicate that elevated plasma concentrations of Gal-3 are indeed a significant risk factor for MCI in T2DM patients, with an OR of 1.954 (95% CI: 1.566–2.438, *p* < 0.001). Moreover, after adjusting for age, gender, education, DM duration, and the prevalence of hypertension, the association between higher levels of Galectin-3 and increased risk of MCI remained significant, with an adjusted OR of 2.028 (95% CI: 1.570–2.620, *p* < 0.001; Table 4). Specifically, for each unit increase in Gal-3 levels (1 ng/mL), the risk of MCI increased by approximately 2 times. Furthermore, the subgroup analysis indicated that although Galectin-3 influenced the incidence of MCI across various clinical factors, there were no significant differences between the subgroups (Figure 3). This consistency across diverse demographic and clinical contexts underscores Galectin-3’s potential as a universal biomarker for MCI risk.

**Table 4:** Univariate and multivariate logistic regression analysis of MCI risk among T2DM patients in the training cohort.

Characteristics	Total (*N*)	Univariate analysis		Multivariate analysis	
		Odds ratio (95% CI)	*p*-value	Odds ratio (95% CI)	*P*-value
Age (year)	231	1.099 (1.059–1.140)	<0.001	1.107 (1.058–1.158)	<0.001
Gender	231				
Male	145	Reference		Reference	
Female	86	1.617 (0.944–2.771)	0.08	1.028 (0.530–1.994)	0.935
Education (year)	231	0.846 (0.770–0.930)	<0.001	0.892 (0.799–0.997)	0.044
DM Duration (year)	231	1.045 (1.006–1.087)	0.024	1.001 (0.953–1.050)	0.975
HTN	231				
No	111	Reference		Reference	
Yes	120	1.835 (1.083–3.110)	0.024	0.961 (0.387–2.386)	0.932
HTN Duration (year)	231	1.032 (1.002–1.062)	0.037	0.989 (0.941–1.040)	0.672
Galectin-3 (ng/ml)	231	1.954 (1.566–2.438)	<0.001	2.028 (1.570–2.620)	<0.001

In summary, the independent contribution of Gal-3 to MCI risk became more pronounced after adjusting for other confounding factors, indicating that Gal-3 has a significant impact on MCI risk independent of other variables.

### 3.4 Construction and evaluation of nomogram scoring system

**Figure 4:**
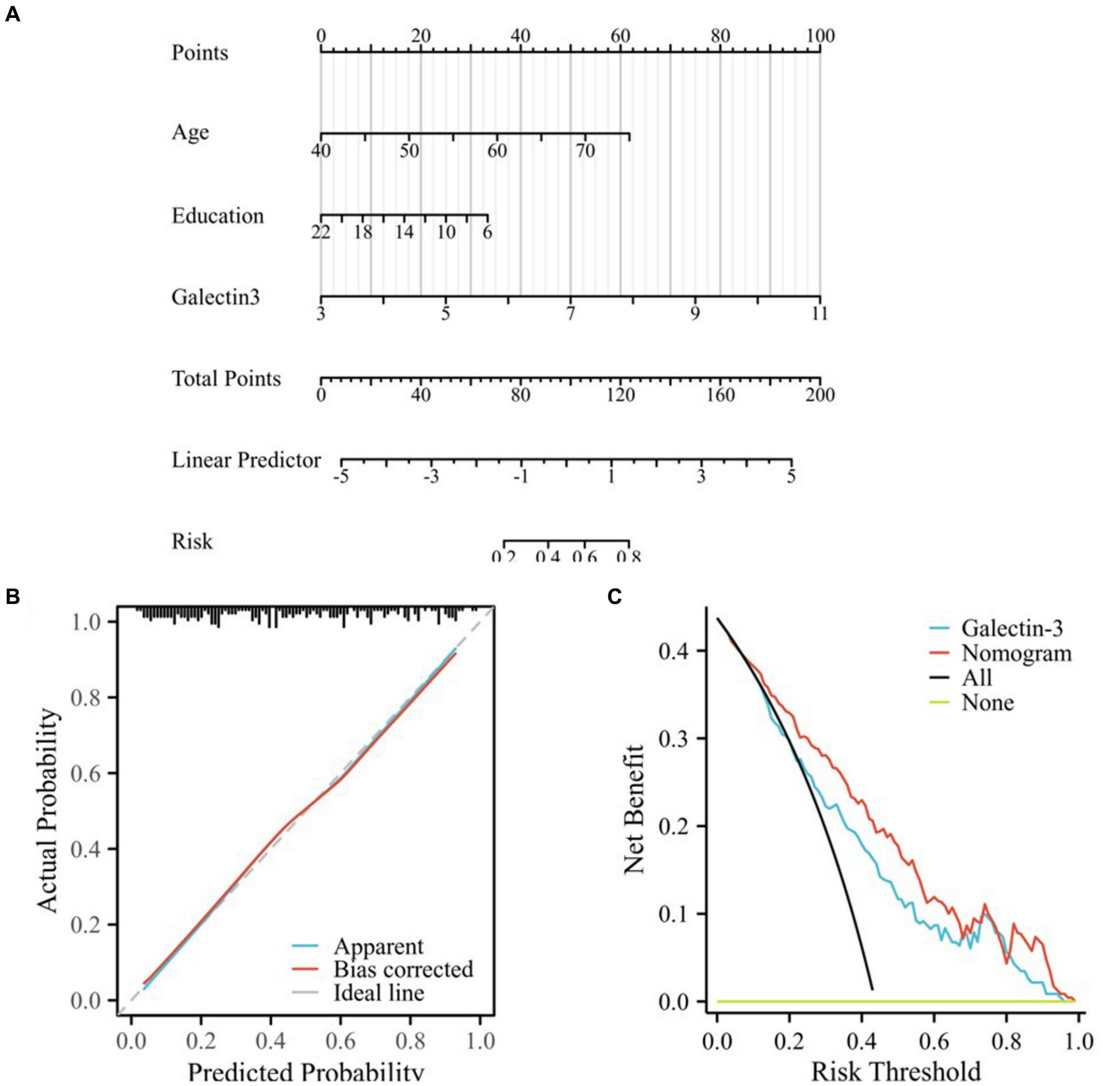
Calibration and clinical use of the nomogram for predicting MCI in T2DM. **(A)** Developed nomogram for predicting MCI in T2DM, created from the training cohort. “Points” referred to the points assigned to individual risk factors, which were summed to yield the “Total points.” “Risk” was calculated based on the “Total points.” **(B)** Calibration curves for internal validation of the nomogram. Ideal line representing the ideal prediction, apparent line representing the predictiveness curves, bias-corrected line representing the calibration curve, the bias-corrected line was close to the ideal line, which indicated that the nomogram was well calibrated. **(C)** DCA curves of the diagnostic nomogram. The clinical benefit and the scope of applications of the nomogram, evaluated by the DCA curves. The x-axis represented the risk threshold probability which ranged from 0 to 1. The y-axis showed the calculated net benefit corresponding to a given threshold probability. “All” represented net benefit of intervening all patients, and “None” meant net benefit of no patient with intervention, whose net gain was zero.

In this study, a nomogram and novel scoring system were established by combining age, Gal-3 levels, and educational levels to predict MCI in patients with T2DM based on regression analysis and clinical high-risk factors, as shown in Figure 4A. All risk factors were assigned numeric scores for quantification, and the final risk score was calculated by summing up the scores of each item using the nomogram depicted in Figure 4A. This allowed us to predict MCI in T2DM patients according to the total points of all risk factors.

The calibration plots demonstrated excellent concordance between the predictions generated by the nomogram and the observed outcomes (Figure 4B). The optimism-corrected C-index to assess the discrimination of the nomogram model was 0.813 (95% CI: 0.757–0.868), indicating accurate and robust performance. Calibration plots showed that the nomogram predictions agreed well with actual observations, as validated by the Hosmer-Lemeshow goodness-of-fit test (χ2 =  2.1427, *P =* 0.9763), indicating no significant difference between the predicted and observed values and confirming that the model had a good fit.

Decision curve analysis (DCA) was employed to evaluate the clinical applicability of the diagnostic nomogram (Figure 4C). The net benefit of DCA was determined by subtracting the proportion of false-positive patients from the proportion of true-positive patients. The decision curve demonstrated a greater net benefit of using nomogram-based MCI risk estimates to determine whether to recommend screening compared to the strategies of screening all or no patients.

Within the probability range of predicting MCI from 0.07 to 1.0, the nomogram provided more benefit compared to the “screen all patients” or the “screen no patients” schemes. When the threshold probability was 0.43, T2DM patients with MCI would benefit the most from using this nomogram. If the threshold probability was greater than 0.07, using the nomogram to predict MCI proved more beneficial in most cases compared to using the single indicator, Gal-3, and from a threshold of 0.13 to about 0.85, the nomogram model consistently outperformed the Gal-3 model in net benefit, demonstrating superior performance especially in scenarios requiring a balance between false positives and false negatives (as shown in Figure 4C; Supplementary material).

Our analysis indicated that if the threshold probability was greater than 0.07, using this nomogram to predict MCI in T2DM patients would provide the most benefit among all models. Specifically, the nomogram surpassed the performance of Gal-3, indicating that the constructed nomogram was a reliable scoring system.

### 3.5 Evaluating nomogram performance for MCI prediction in T2DM

**Figure 5:**
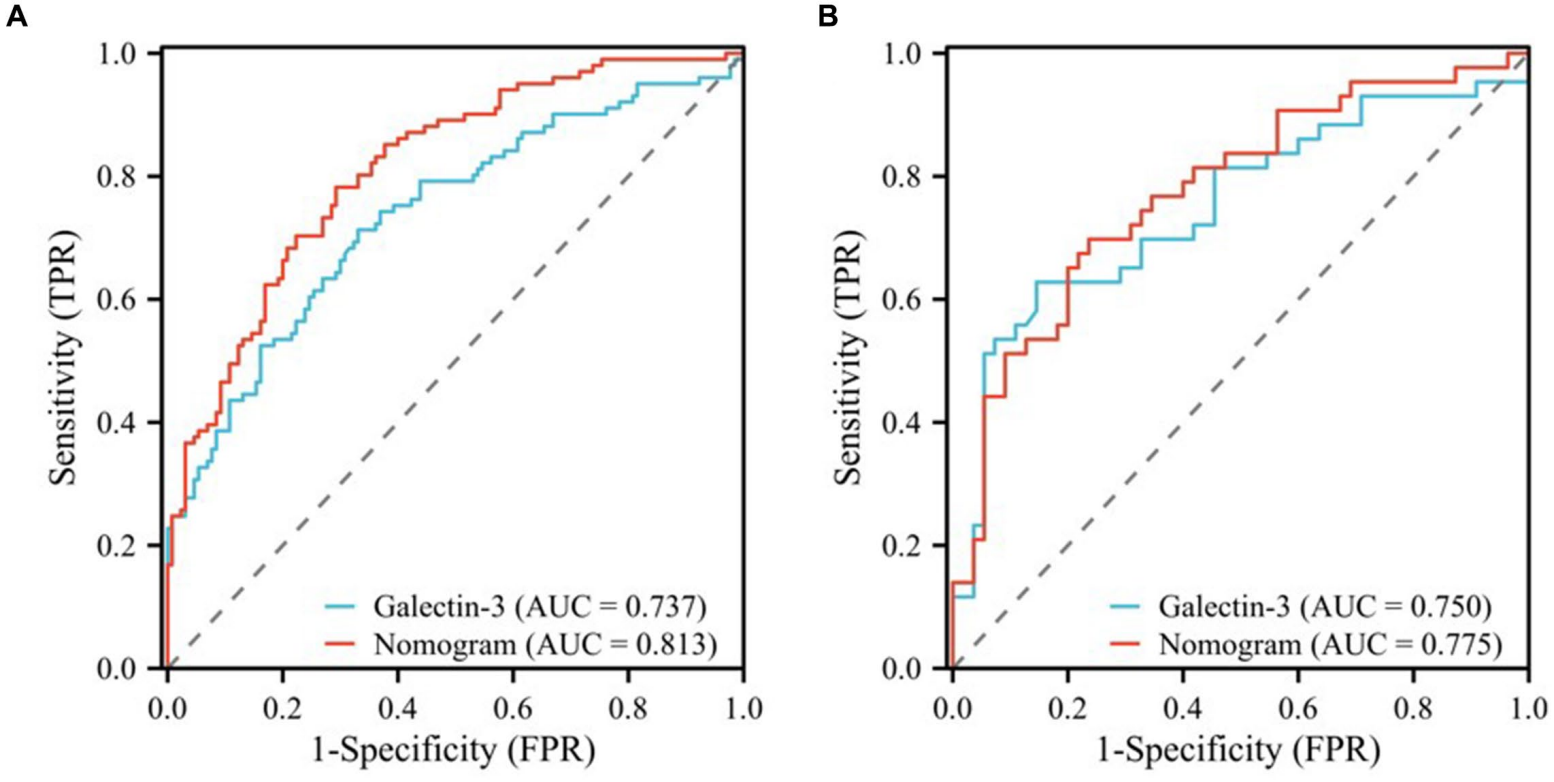
ROC curves of the galectin-3 and nomogram models for the training **(A)** and validation **(B)** cohorts.

In this study, we evaluated the predictive efficacy of the galectin-3 and nomogram models for MCI in patients with T2DM. The Area Under the Curve (AUC) values, detailed in Table 5, and the Receiver Operating Characteristic (ROC) curves, depicted in Figure 5, served as our primary metrics. Figure 5A shows the ROC curves for the training cohort. Here, the galectin-3 model achieved an AUC of 0.737, while the nomogram model demonstrated a superior AUC of 0.813, which was statistically significant (*p* = 0.002), indicating robust predictive power (Table 5). Figure 5B, displaying the validation cohort, reveals that the galectin-3 model achieved an AUC of 0.750, and the nomogram model slightly outperformed it with an AUC of 0.775.

**Table 5:** The AUC of galectin-3 and nomogram for predicting MCI in T2DM.

Models	Training cohort (*N* = 231)		Validation cohort (*N* = 98)	
	AUC (95% CI)	*P*-value	AUC (95% CI)	*P*-value
Galectin-3	0.737(0.671–0.803)	0.002	0.750(0.648–0.853)	0.331
Nomogram	0.813(0.758–0.867)		0.775(0.681–0.870)	

To ascertain the statistical significance of the performance differences between these models, a DeLong test was performed. These results underscore the effectiveness of both models in predicting MCI, with the nomogram model consistently showing slightly superior performance across both the training and validation cohorts.

### 3.6 Prediction of microRNA targets in galectin-3 regulation

The upstream regulatory microRNAs of LGALS3 were predicted using multiple databases: miRWalk which catalogs both predicted and validated miRNA binding sites across the entire gene sequence, GeneCards, miRBase, miRTarBase, and TargetScan. Additionally, miRBase and miRTarBase provide information about experimentally validated miRNA-mRNA interactions, offering a comprehensive resource for understanding miRNA regulation of LGALS3. Three intersecting microRNAs, specifically hsa-miR-128-3p targeting the 3′-UTR, and hsa-miR-424-3p and hsa-miR-744-5p targeting the coding sequence (CDS) of the LGALS3 gene, were identified (Supplementary Figure 3; Supplementary Table 2).

### 3.7 Identification of intersecting genes levels in T2DM with MCI

**Figure 6:**
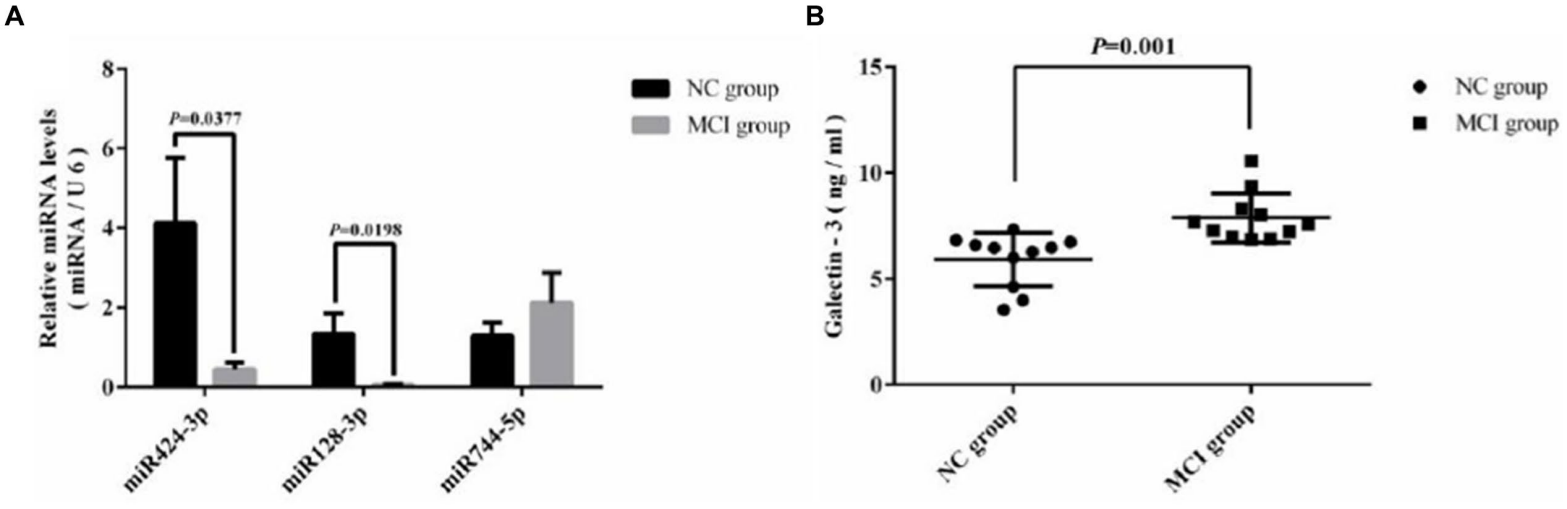
Comparative analysis of expression levels. **(A)** miRNA expression in two groups measured via RT-PCR; **(B)** Galectin-3 expression in two groups assessed by ELISA.

**Figure 7:**
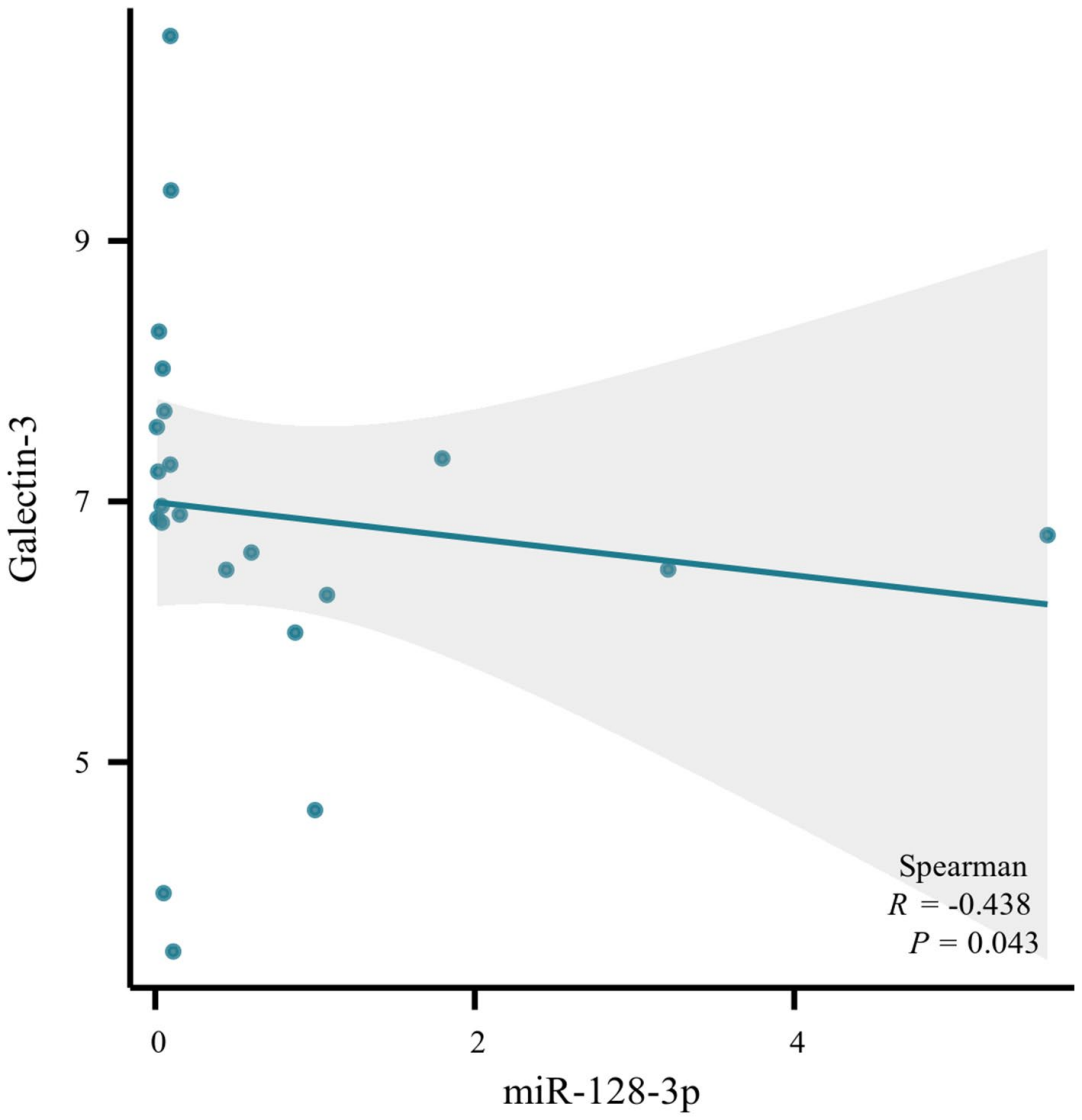
Scatter plot of correlation analysis between galectin-3 and miR-128-3p.

Quantitative real-time PCR (qRT-PCR) assays were conducted to measure the levels of miRNA128, miRNA424, hsa-miR-744, and LGALS3 in the peripheral blood mononuclear cells (PBMCs) from blood samples of 22 randomly selected subjects, with their clinical information detailed in Supplementary Table 3. Compared with the NC group, hsa-miR-128-3p, hsa-miR-424-3p in MCI group tended to be downregulated, while Galectin-3 tended to be upregulated, with all differences reaching statistical significance. The relative expression trends were consistent with bioinformatics analysis. However, hsa-miR-744 showed an increase, with no significant statistical difference observed (Figure 6). Further analysis using Spearman’s correlation showed a significant negative association between Gal-3 levels and miR-128-3p (R = −0.438, *p* = 0.043), as illustrated in Figure 7.

### 3.8 Result of luciferase activity assay

**Figure 8:**
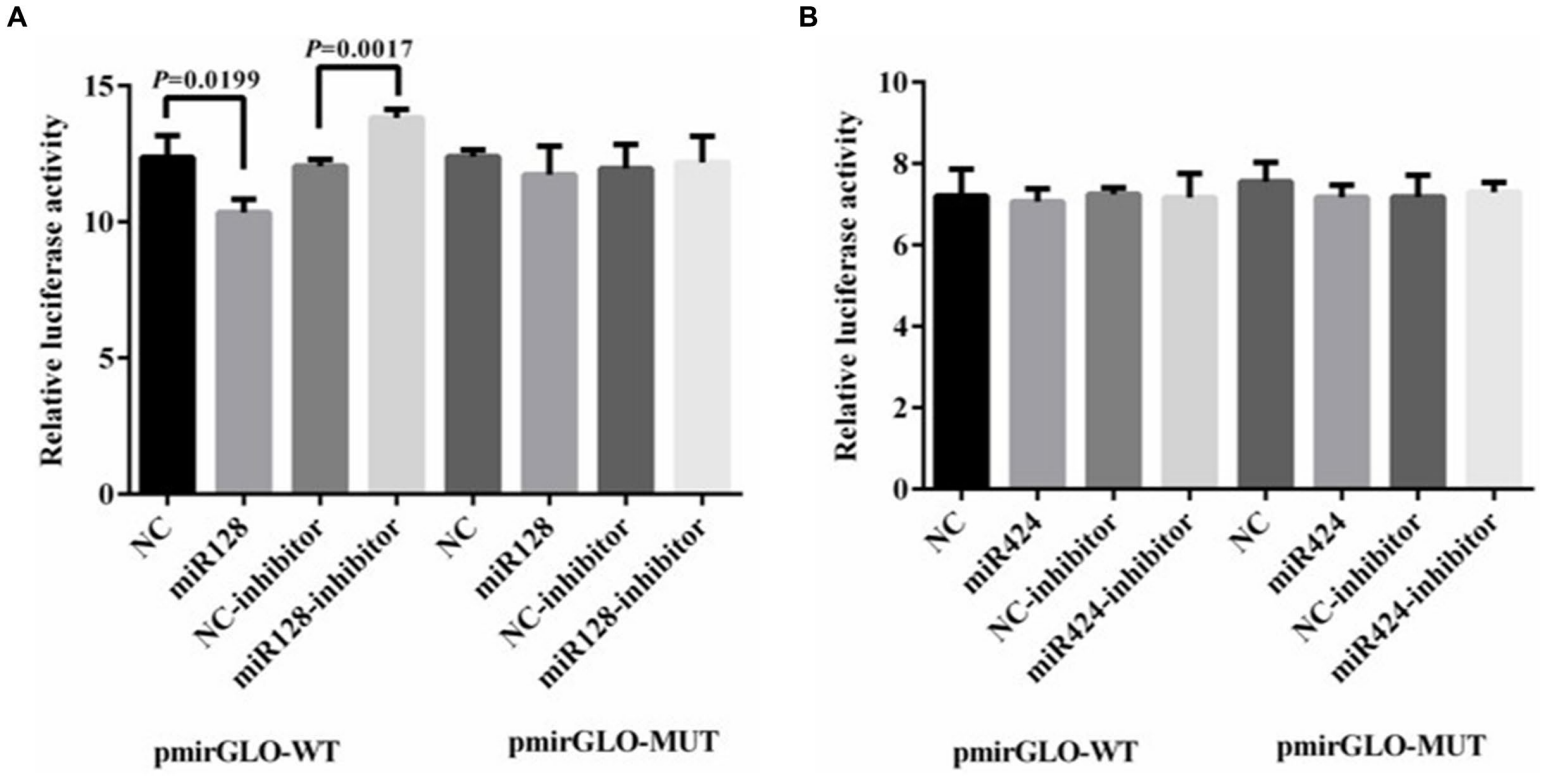
Result of luciferase activity assay. **(A)** Luciferase reporter assay was performed in HEK 293T cells transfected with LGALS3 wild (or mutant) type and miR-128 mimics (or inhibitor); **(B)** Luciferase reporter assay was performed in HEK 293T cells transfected with LGALS3 wild (or mutant) type and miR-424 mimics (or inhibitor); NC, mimic control; NC-inhibitor, inhibitor control.

To determine whether miRNAs directly target LGALS3, luciferase reporter assays were performed by cotransfecting HEK293 cells with either hsa-miR-128-3p mimics/inhibitors and a luciferase reporter vector containing the LGALS3 3′-UTR binding site, or with hsa-miR-424-3p mimics/inhibitors along with a reporter vector that includes the LGALS3 CDS binding site. The results from the dual luciferase reporter gene assay indicated that the luciferase activity of the LGALS3 wild type was significantly reduced following transfection with the miR-128-3p mimic, whereas the luciferase activity of the LGALS3 mutant remained unchanged (Figure 8A). This suggests that miR-128-3p can specifically bind to the wild type LGALS3. To further investigate the regulatory role of miR-128-3p on LGALS3, the luciferase activity of the LGALS3 wild type was upregulated upon transfection with the miR-128-3p inhibitor (Figure 8A). No changes in luciferase activity were observed in the LGALS3 mutant type under these conditions. These findings demonstrate that LGALS3 could be targeted and downregulated by miR-128-3p. In contrast, transfection with either miR-424-3p mimics or inhibitors did not alter the luciferase activity in both the wild-type and mutant LGALS3 plasmids (Figure 8B), indicating that miR-424-3p could not regulate LGALS3.

## 4 Discussion

Diabetes mellitus (DM) is recognized as a significant risk factor for Alzheimer’s disease (AD), with studies reporting that T2DM increases the risk of dementia by nearly 37% and the risk of MCI by about 20% (5). Since there are currently no effective treatments for AD, and considering that MCI serves as a transitional stage between normal aging and dementia with subtle cognitive deficits, timely interventions could potentially prevent disease progression and even restore normal cognitive functions (39, 40). In recent years, galectin-3 (Gal-3), a member of the galectin family, has garnered considerable attention for its role in neuroinflammation and neurodegeneration (41). Studies have shown that Gal-3 significantly contributes to the proinflammatory response in neurodegenerative disorders, thereby exacerbating cognitive decline (42–44). Research on the effects of miRNAs in regulating LGALS3 gene, particularly Galectin-3, in human neurobiology is limited. Earlier studies, mainly in murine models, focus on disorders like ADHD and Alzheimer’s, demonstrating how miRNAs such as microRNA let-7d and miR-155 regulate Galectin-3, potentially offering new therapeutic insights (45, 46).

Our study explored the regulatory role of miRNAs on LGALS3 and their potential impact on cognitive impairment in T2DM patients. Bioinformatics analysis identified three miRNAs—hsa-miR-128-3p, hsa-miR-424-3p, and hsa-mir-744-5p—that might target the LGALS3 gene. However, qRT-PCR assays revealed that hsa-miR-128-3p and hsa-miR-424-3p were downregulated in MCI patients compared to normal controls, while Gal-3 was upregulated. Further analysis indicated a significant negative correlation between Gal-3 and miR-128-3p, despite the small sample size. Results from luciferase reporter assays further support this, showing that miR-128-3p could specifically bind and downregulate LGALS3, whereas miR-424-3p did not exhibit similar regulatory effects. This inverse relationship has suggested that the downregulation of miR-128-3p led to increased Gal-3 levels, potentially contributing to cognitive decline. Previous study has also shown that hsa-miR-128-3p was downregulated in individuals with low cognitive performance and might be a blood biomarker for subclinical cognitive deficits in Alzheimer’s disease (47). miRNAs, as biomarkers, could be obtained from cerebrospinal fluid, plasma, and peripheral blood mononuclear cells (PBMCs), with peripheral miRNAs mirroring changes similar to central miRNAs (22, 48), thus indirectly reflecting the trends in Gal-3 expression. Targeting Gal-3 with hsa-miR-128-3p could be key for managing diabetic cognitive dysfunction, positioning it as a crucial target for novel MCI strategies in T2DM. To substantiate this approach, further foundational research and an expansion of sample sizes are imperative.

Our analysis further revealed that T2DM patients with MCI exhibited significantly higher levels of Gal-3 in both training and validation cohorts, aligning with studies suggesting that serum Gal-3 levels might be a new risk factor for MCI in type 2 diabetes mellitus patients and could serve as a potential target for therapeutic interventions aimed at preventing cognitive impairment in diabetes (49, 50). Further correlation analyses, adjusted for age, gender, education, prevalence of hypertension, and duration of DM, indicated that elevated Gal-3 levels were associated with poorer cognitive performance, as evidenced by lower scores on neuropsychometric tests (MOCA, MMSE, DST) and higher TMTB scores, which reflect diminished global cognitive and executive functions. These findings might suggest that Gal-3 was not merely a biomarker for cognitive impairment in T2DM patients but could also be a viable therapeutic target. Additionally, Gal-3 derived from mesenchymal stem cells has been shown to clear aberrant forms of tau and reduce hyperphosphorylation of tau both *in vitro* and *in vivo*, as well as ameliorate deficits in spatial learning and memory, confirming the potential therapeutic role of Gal-3 in AD pathology and associated memory impairment (51). In previous studies, elevated levels of Gal-3 have been observed not only in the brain parenchyma and cerebrospinal fluid (CSF) but also in the serum of AD patients (52–54). This increase is often associated with the degradation of the blood–brain barrier (BBB) observed during the progression of AD (55), potentially leading to elevated peripheral blood levels of Gal-3. Furthermore, serum Gal-3 levels significantly paralleled the severity of memory loss (56) and the stage of AD (57). Such findings suggest that peripheral blood levels of Gal-3 could reflect inflammatory states within the brain, although their accuracy may be influenced by the integrity of BBB. Building on our findings, logistic regression analyses further identified Gal-3 levels, along with age and education, as independent predictors of MCI. Utilizing these predictors, a nomogram was developed and subsequently validated for its predictive accuracy. The nomogram demonstrated excellent calibration and discrimination, achieving an AUC of 0.813 in the training cohort and 0.775 in the validation cohort, surpassing the performance of the Gal-3 model alone. DCA also substantiated the clinical utility of the nomogram by illustrating a higher net benefit for predicting MCI in T2DM patients compared to relying solely on Gal-3 levels. This tool offers clinicians a robust method for assessing the risk of MCI, facilitating informed decision-making regarding early intervention strategies. Additionally, Modified Citrus Pectin (MCP), a classical carbohydrate-based Gal-3 inhibitor, has demonstrated efficacy in T2DM rats to improve insulin sensitivity, attenuate memory impairment, and inhibit oxidative stress and neuroinflammation (50) and Gal-3’s deletion could attenuate Aβ oligomerization -mediated inflammatory response (56). Specifically, MCP prevented BBB disruption in mouse subarachnoid hemorrhage by inhibiting Gal-3 (58), thus modulating the inflammatory responses linked to this condition. Underscoring the potential of developing BBB-permeable selective Gal-3 inhibitors. These inhibitors could then be further tested for therapeutic efficacy in brain disease models, representing a significant potential in the treatment of AD and cognitive disorders. Although our study did not observe lipid level differences between MCI group and NC group, lipid lowering therapy is beneficial in reducing cardiovascular risks for T2DM patients, particularly for women (59). However, the impact on cognitive impairment varies, with some studies suggesting no improvement in cognitive functions (60, 61), thus warranting further investigation. Furthermore, insulin resistance in T2DM may exacerbate oxidative stress, contributing to the neurodegeneration associated with AD (62), suggesting that antioxidants (63) could play a significant role in mitigating cognitive decline in T2DM patients.

This study is subject to several limitations. Firstly, it relied on retrospective data, necessitating validation through prospective studies and the low proportion of female participants in our study might have limited the applicability of our findings concerning the impact of gender differences in galectin-3 levels on MCI risk. Future research will aim to address this by increasing the sample size. Despite local expression variations of galectin-3 suggesting potential differences in specific disease states, no significant differences were observed in peripheral blood. Additionally, our clinical data lacked neuroimaging and cerebrospinal fluid analyses. Finally, the absence of multicenter validation was a critical gap. Although the nomogram proposed in this paper was evaluated and performed well within an internal validation cohort, further validation by other centers was essential to assess the reliability of our predictive nomogram. Consequently, multicenter validation of this scoring system with a large study population is urgently required to provide robust evidence for its future clinical application.

In summary, our study underscores the value of Gal-3 as a biomarker for cognitive impairment in T2DM and presents a validated nomogram that integrates Gal-3 levels, age, and education to predict MCI. This model offers a practical approach for early detection and management of cognitive decline in T2DM patients, facilitating timely therapeutic interventions.

## 5 Conclusion

Galectin-3 was identified as an independent risk factor for MCI in patients with T2DM, significantly correlating with diabetic cognitive dysfunction. The predictive nomogram based on Gal-3 levels has been developed to support clinical decision-making for T2DM patients at risk of MCI. Our findings underscore the need for large, multi-center, prospective studies to validate these results and refine the model for broader applicability. Moreover, Gal-3, as a potential risk molecule involved in the development of MCI, plays a crucial role in facilitating effective interventions or delaying diabetic cognitive impairment progression. Our research provides a foundation for future studies focused on customizing interventions for diabetic cognitive dysfunction.

## Data availability statement

The original contributions presented in the study are included in the article/Supplementary material, further inquiries can be directed to the corresponding author.

## Ethics statement

The studies involving humans were approved by the Research Ethics Committee, Affiliated ZhongDa Hospital of Southeast University (approval no. of ethics committee: 2023ZDSYLL435-P01). The studies were conducted in accordance with the local legislation and institutional requirements. The participants provided their written informed consent to participate in this study. Written informed consent was obtained from the individual(s) for the publication of any potentially identifiable images or data included in this article.

## Author contributions

XZ: Conceptualization, Data curation, Formal analysis, Investigation, Methodology, Project administration, Resources, Software, Supervision, Validation, Visualization, Writing – original draft, Writing – review & editing. ND: Methodology, Project administration, Software, Validation, Visualization, Writing – review & editing. DY: Data curation, Supervision, Validation, Writing – review & editing. TN: Data curation, Supervision, Writing – review & editing. SW: Conceptualization, Funding acquisition, Project administration, Supervision, Writing – review & editing.
