# Supplementary material for: Exploring galectin-3’s role in predicting mild cognitive impairment in type 2 diabetes and its regulation by miRNAs
Source: Front Med (Lausanne). 2024 Jul 31;11:1443133. doi: 10.3389/fmed.2024.1443133 (PMC11322075; doi:10.3389/fmed.2024.1443133)
Supplement: Supplementary file 2 [file Data_Sheet_1.docx]

**supplementary materials**

**
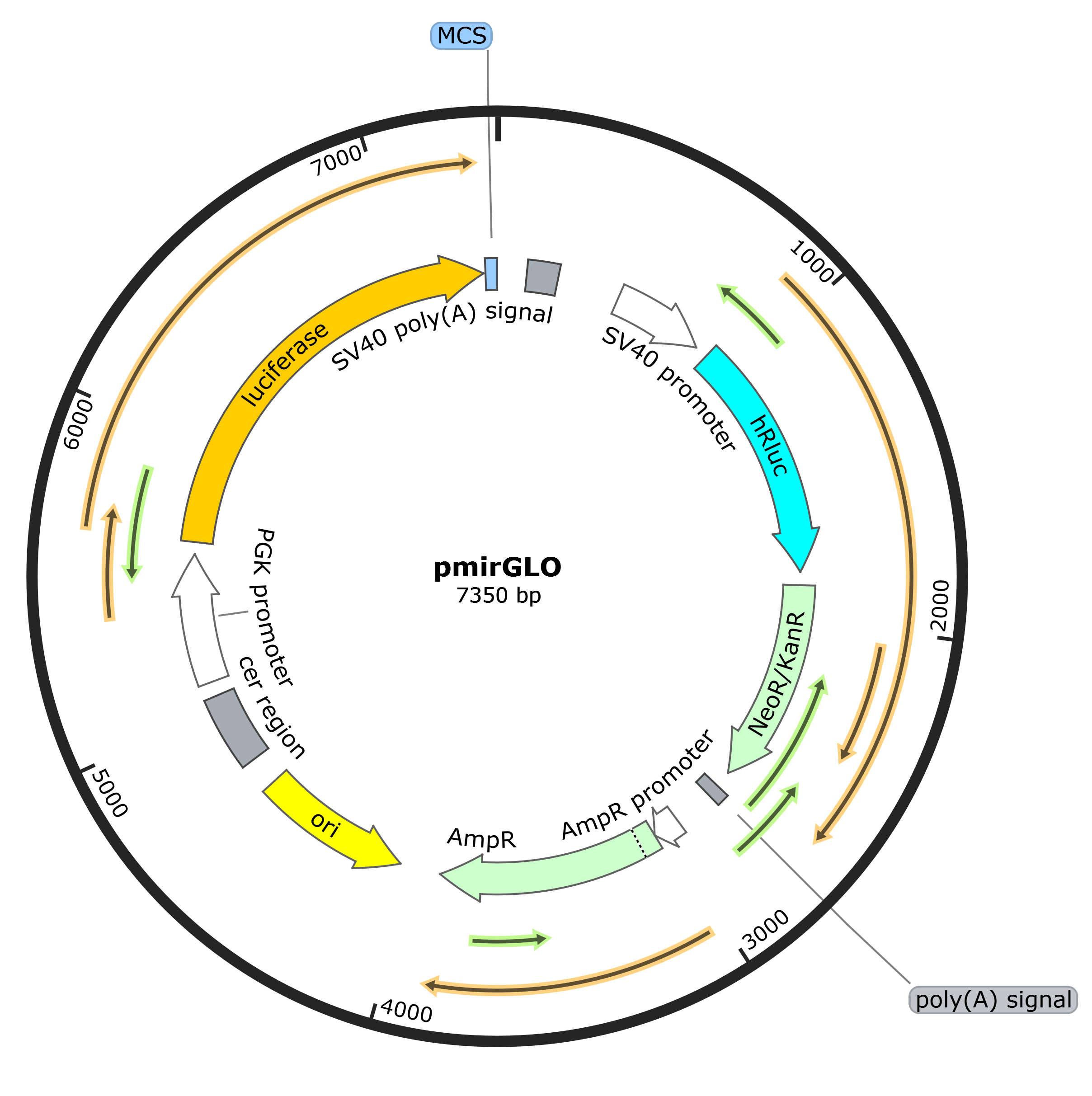
**

**Supplementary Figure 1. Structural Diagram of the pmirGLO Vector**

**
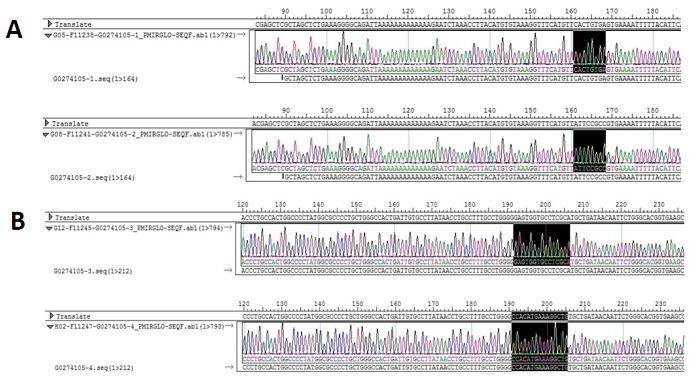
**

**Supplementary Figure 2. A:Partial Results of Sequencing Comparisons for LGALS3 (MRE128-3p wt) Wild-Type and LGALS3 (MRE128-3p mut) Mutant Recombinant Vectors**

**B:Partial Results of Sequencing Comparisons for LGALS3 (MRE424-3p wt) Wild-Type and LGALS3 (MRE424-3p mut) Mutant Recombinant Vectors**

**
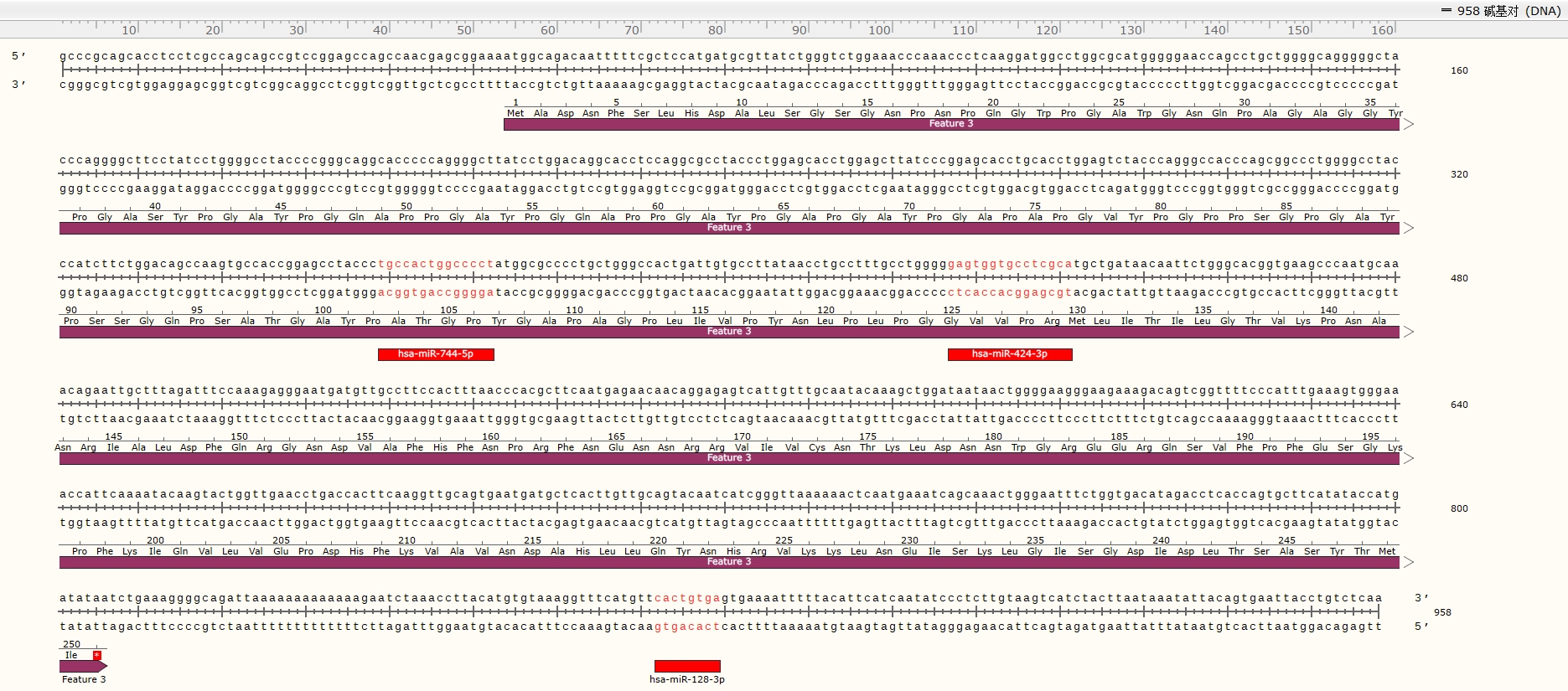
**

**Supplementary Figure 3. miRNA Binding Sites on the LGALS3 Gene**

**Supplementary Table 1. Association Between Galectin-3 and Cognitive Function in T2DM Patients in the Training Cohort**

|  | Model 1 | |  | Model 2 | |  | Model 3 | |
| --- | --- | --- | --- | --- | --- | --- | --- | --- |
| Variable | R coefficient | P value |  | R coefficient | P value |  | R coefficient | P value |
| MOCA | -0.515 | 0.000 |  | -0.547 | 0.000 |  | -0.542 | 0.000 |
| MMSE | -0.345 | 0.000 |  | -0.382 | 0.000 |  | -0.368 | 0.000 |
| DST | -0.308 | 0.000 |  | -0.315 | 0.000 |  | -0.309 | 0.000 |
| VFT | -0.208 | 0.002 |  | -0.157 | 0.018 |  | -0.159 | 0.018 |
| CDT | -0.185 | 0.005 |  | -0.198 | 0.003 |  | -0.193 | 0.004 |
| TMTA | 0.074 | 0.267 |  | 0.041 | 0.545 |  | 0.013 | 0.852 |
| TMTB | 0.202 | 0.002 |  | 0.257 | 0.000 |  | 0.256 | 0.000 |
| AVLT-IR | -0.114 | 0.089 |  | -0.121 | 0.074 |  | -0.138 | 0.042 |
| AVLT-DR | -0.091 | 0.186 |  | -0.094 | 0.178 |  | -0.091 | 0.193 |
| LMT | -0.017 | 0.800 |  | -0.001 | 0.991 |  | -0.010 | 0.883 |

Annotation:

Model 1 showed the Spearman association between Galectin-3 and cognitive preference test scores;

Model 2 showed the partial association between Galectin-3 and cognitive preference test scores adjusted for age, gender and education;

Model 3 showed the partial association between Galectin-3 and cognitive preference test scores adjusted for age, gender, education, the prevalence of Hypertension and DM Duration.

**Supplementary Table 2. Bioinformatic analysis predicted that the 3'UTR or CDS of LGALS3 contained binding site sequences for miRNA**

| **Target gene and miRNA** | **The binding site sequences** |
| --- | --- |
| Position 66-73 of LGALS3 3' UTR | 5'...UAAAGGUUUCAUGUUCACUGUGA...3' |
| hsa-miR-128-3p | 3'...UUUCUCUGGCCAAGUGACACU...5' |
| Position 427-441 of LGALS3 CDS | 5'...AGTGGTGCCTCGCA...3 |
| hsa-miR-424-3p | 3'...TATCGTCGCGGAGTGCAAAAC...5' |
| Position 359-372 of LGALS3 CDS | 5'...GCCACTGGCCCCT...3' |
| hsa-miR-744-5p | 3'...ACGACAATCGGGATCGGGGCGT...5' |

Annotation:Sequences displayed include both RNA and DNA types. DNA sequences, sourced from miRWalk, predict miRNA binding sites, while RNA sequences from TargetScan detail regions complementary to the target miRNAs.The estimated binding sites are shown in red.

**Supplementary Table 3. Clinical Data of Participants Involved in the qRT-PCR Experiment**

| Characteristics | NC group | MCI group | P value |
| --- | --- | --- | --- |
| n | 11 | 11 |  |
| Age(year) | 56.091 ± 11.229 | 59.636 ± 6.9609 | 0.386 |
| Gender, n (%) |  |  | 1 |
| Male | 9 (40.9%) | 8 (36.4%) |  |
| Female | 2 (9.1%) | 3 (13.6%) |  |
| Education | 12 (11.5, 14) | 9 (9, 11.5) | 0.02 |
| DM Duration (year) | 14.273 ± 7.9005 | 12.727 ± 7.5907 | 0.645 |
| HTN, n (%) |  |  | 1 |
| No | 6 (27.3%) | 5 (22.7%) |  |
| Yes | 5 (22.7%) | 6 (27.3%) |  |
| HTN Duration | 0 (0, 7.5) | 5 (0, 15) | 0.46 |
| Smoking history, n (%) |  |  | 0.361 |
| No | 9 (40.9%) | 6 (27.3%) |  |
| Yes | 2 (9.1%) | 5 (22.7%) |  |
| Alcohol use, n (%) |  |  | 1 |
| No | 10 (45.5%) | 9 (40.9%) |  |
| Yes | 1 (4.5%) | 2 (9.1%) |  |
| BMI(Kg/m2) | 24.773 ± 3.0143 | 25.414 ± 2.6425 | 0.602 |
| HbA1c(%) | 9.6309 ± 2.3124 | 8.7745 ± 1.8101 | 0.345 |
| FBG(mmol/L) | 7.27 (6.53, 8.77) | 7.61 (6.43, 8.36) | 0.898 |
| TG(mmol/L) | 1.48 (1.035, 2.185) | 1.79 (1.26, 1.985) | 0.818 |
| TC(mmol/L) | 4.7327 ± 1.209 | 4.5173 ± 1.4618 | 0.71 |
| Cr(µmol/L) | 71.727 ± 26.766 | 70 ± 20.596 | 0.867 |
| BUN(mmol/L) | 6.3636 ± 1.6984 | 5.8455 ± 1.6537 | 0.477 |
| ALT(U/L) | 18 (16, 25.5) | 17 (14.5, 21.5) | 0.576 |
| AST(U/L) | 16 (13.5, 22.5) | 19 (16.5, 20) | 0.553 |
| MOCA | 28 (28, 28.5) | 24 (22, 24.5) | < 0.001 |
| MMSE | 30 (28.5, 30) | 26 (25, 28) | 0.001 |
| DST | 12.7 ± 2.5841 | 10.636 ± 3.5006 | 0.144 |
| VFT | 17.5 ± 3.4721 | 14.364 ± 4.7806 | 0.105 |
| CDT | 4(2, 4) | 2 (2, 4) | 0.116 |
| TMTA | 51 (46.25, 54.5) | 71 (64, 76) | 0.002 |
| TMTB | 153.9 ± 30.989 | 203.09 ± 68.073 | 0.048 |
| AVLT-IR | 14.3 ± 5.3344 | 15.364 ± 5.8698 | 0.67 |
| AVLT-DR | 6.1 ± 4.6774 | 5.0909 ± 2.5477 | 0.541 |
| LMT | 4 (3, 9.25) | 4 (2.5, 6.5) | 0.644 |
